# The Shaping of Two Distinct Dendritic Spikes by A-Type Voltage-Gated K^+^ Channels

**DOI:** 10.3389/fncel.2015.00469

**Published:** 2015-12-09

**Authors:** Sungchil Yang, Cha-Min Tang, Sunggu Yang

**Affiliations:** ^1^Department of Biomedical Sciences, City University of Hong KongKowloon, Hong Kong; ^2^Department of Neurology and Department of Physiology, University of Maryland School of MedicineBaltimore VAMC, MD, USA; ^3^Department of Nano-Bioengineering, Incheon National UniversityIncheon, South Korea

**Keywords:** CA1 pyramidal neuron, voltage-gated calcium channels, A-type K^+^ channels, dendritic integration, dendritic excitability

## Abstract

Dendritic ion channels have been a subject of intense research in neuroscience because active ion channels in dendrites shape input signals. Ca^2+^-permeable channels including NMDA receptors (NMDARs) have been implicated in supralinear dendritic integration, and the I_A_ conductance in sublinear integration. Despite their essential roles in dendritic integration, it has remained uncertain whether these conductance coordinate with, or counteract, each other in the process of dendritic integration. To address this question, experiments were designed in hippocampal CA1 neurons with a recent 3D digital holography system that has shown excellent performance for spatial photoactivation. The results demonstrated a role of I_A_ as a key modulator for two distinct dendritic spikes, low- and high-threshold Ca^2+^ spikes, through a preferential action of I_A_ on Ca^2+^-permeable channel-mediated currents, over fast AMPAR-mediated currents. It is likely that the rapid kinetics of I_A_ provides feed-forward inhibition to counteract the regenerative Ca^2+^ channel-mediated dendritic excitability. This research reveals one dynamic ionic mechanism of dendritic integration, and may contribute to a new understanding of neuronal hyperexcitability embedded in several neural diseases such as epilepsy, fragile X syndrome and Alzheimer’s disease.

## Introduction

Ion channels in dendrites play a determinant role in shaping the response pattern of a neuron (Catterall, 1998; Larkum et al., 1999b, 2009; Gasparini et al., 2004; Frick and Johnston, 2005; Major et al., 2008, 2013; Ginger et al., 2013; Palmer et al., 2014; Wang et al., 2014; Yang et al., 2014a). They initiate and modify an input signal through operations such as filtering, amplification and gain-control, to optimally code information. There are abundant A-type K^+^ channels (I_A_) and Ca^2+^-permeable channels in the dendrites (Frick et al., 2003; Cai et al., 2004; Kim et al., 2007; Jung et al., 2008; Wang et al., 2014). The rapid activating and inactivating nature of I_A_ has a crucial role in shaping the input signals in the dendrite. Backpropagating action potentials (bAPs) decrease in amplitude with distance from the soma and often failed to invade distal dendrites (Spruston et al., 1995; Golding et al., 2002; Frick et al., 2003), and this amplitude decay is attributed in part to high expression of I_A_ in distal locations (Hoffman et al., 1997; Grewe et al., 2010). It is also considered that I_A_ plays a role in dampening local dendritic Na^+^ spikes of CA1 pyramidal neurons (Gasparini et al., 2004). Consistently, loss of Kv4.2, one of the channel subunits contributing to I_A_, increases the potentials generated in dendrites, breaking down the normal electrical compartmentalization of dendrites (Cai et al., 2004). Therefore it has been suggested that I_A_ does not only limit the spread of bAPs, but also actively suppresses the anterograde propagation of local Na^+^ and Ca^2+^ signals generated in thin branches (Losonczy and Magee, 2006; Losonczy et al., 2008).

While I_A_ has been implicated in sublinear dendritic integration (Urban and Barrionuevo, 1998; Cash and Yuste, 1999; Losonczy and Magee, 2006; Yang et al., 2014a), NMDARs have been proposed to drive supralinear integration within thin dendritic branches (Schiller et al., 2000; Branco and Hausser, 2011; Major et al., 2013; Yang et al., 2014a). Although both are highly expressed on the thin dendrites of pyramidal neurons, a direct interaction between these currents has not been established. Indeed, NMDARs are kinetically slow to activate and inactivate, whereas I_A_ is fast activating and inactivating. NMDA spikes are powerful and difficult to regulate once initiated. The most effective time for their regulation is before they are initiated. In this way, the rapid nature of I_A_ could serve as feed-forward inhibition on NMDAR-mediated dendritic excitability.

While NMDARs are mostly localized in thin dendritic branches, voltage-gated calcium channels (VGCCs) are abundant in apical dendrites (Kim and Connors, 1993; Schiller et al., 1997; Larkum et al., 1999a,b, 2009; Kim et al., 2007). Given the potential difference of NMDARs and VGCCs in dendrites, their contributions to dendrite-to-axon information processing are likely differentiated into generation of three distinct spikes. The information is amplified by (1) NMDA spikes in a distal thin branch where the overwhelming majority of synaptic events occur. It is further magnified in an apical dendrite through (2) VGCC spikes, ultimately propagating into (3) axosomatic sodium spike zone for action potential trigger (Larkum et al., 2009).

We hypothesized that I_A_ counteracts Ca^2+^-permeable channels including NMDARs, thereby affecting expression of dendritic spikes and integration. The results indicate that I_A_ had a more significant action on the slow-acting Ca^2+^-permeable channels than the fast-acting AMPAR-mediated currents in thin branches. Furthermore, the collaborative interaction of I_A_ with Ca^2+^-permeable channels is a critical determinant for shaping dendritic spikes and integration. Here, we propose that I_A_ plays a key role as an internal controller in the Ca^2+^-mediated neuronal excitability.

## Materials and Methods

### Brain Slice and Whole Hippocampus Preparation

All animal handling procedures were approved by the Institutional Animal Care and Use Committee of the University of Maryland and the National Institutes of Health* Guide for the care and use of laboratory animals*, and the Animal Welfare Act (7 U.S.C. et seq.). Sprague-Dawley rats (postnatal age: 4–6 weeks) for brain slices were deeply anesthetized with halothane. The brains were quickly removed and placed into chilled (4°C), oxygenated (5% CO_2_ and 95% O_2_) slicing medium containing (in mM): 212 sucrose, 5 KCl, 1.23 NaH_2_PO_4_, 26 NaHCO_3_, 11 glucose, 1.5 MgCl_2_, 2.5 CaCl_2_. Transverse slices (300–400 μm) were cut using vibratome. Rat Slices were then transferred to a holding chamber containing oxygenated physiological saline made up of (in mM): 124 NaCl, 4 KCl, 1.23 NaH_2_PO_4_, 26 NaHCO_3_, 10 glucose, 1.5 MgCl_2_, 2 CaCl_2_. After at least 1 h recovery, individual slices were transferred to a recording chamber. Oxygenated physiological saline was continuously superfused at a rate of 1.5 ml/min.

### Whole-Cell Patch Recording

Whole-cell patch recordings were obtained using an Axon instruments Axoclamp 700B Amplifier (Molecular Devices), and recording pipettes had tip resistances of 3–7 MΩ when filled with a solution containing (in mM): 135 K-gluconate, 5 KCl, 1 MgCl_2_, 0.02 CaCl_2_, 0.2 EGTA, 10 HEPES, 4 Na_2_-ATP, 0.3 Na-GTP. Alexa 594 (50 μM) was included in the internal solution for visualization. The pH and osmolarity of intracellular solution were adjusted to 7.3 and 290 mOsm, respectively. Recordings were done in current-clamp configuration and cells were held at −65 mV; the resting membrane potential was in the range of −65 ~ −70 mV after whole-cell configuration. During recordings, an access resistance was continually monitored. Recordings were excluded if an input resistance changed by >15%. pClamp Version 10.2 software (Molecular Devices) was used for data acquisition. Whole-cell patch recordings were made on CA1 neurons. Alexa 594 was placed in the patch electrodes and allowed to dialyze into dendritic arbor. Once the fluorescence signal of oblique dendrites became visible, the 3D coordinates for photolysis sites on the dendrites are identified using 3D digital holography.

### 3D Digital Holography with Uncaging

**Figure 1:**
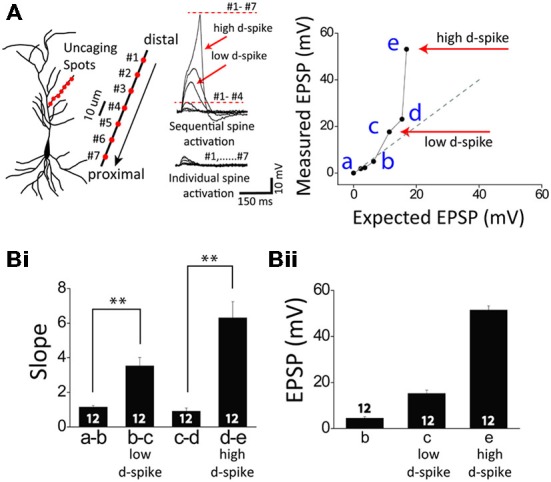
**Two distinct dendritic spikes. (A)** Illustration of a CA1 pyramidal neuron (left) and glutamate uncaging spots (red) / sequential uncaging of distal-to-proximal direction (arrow) in a thin oblique dendrite (right). The representative traces illustrate photoactivated excitatory postsynaptic potentials (EPSPs) in sequential spine activation and individual spine activation. The expected EPSP (gray dot) is an arithmetic addition of the uncaging-induced EPSP of individual spots while the measured EPSP (black line) is the experimentally induced EPSPs corresponding to sequential spot activation. If the slope of the measured EPSP significantly runs over that of the expected EPSP (**Bi**), it is called supralinear summation as the cases of low and high d-spikes. Note there are two distinct dendritic spikes each having a pronounced nonlinear increase: the low-threshold dendritic spike (low d-spike) and the high-threshold dendritic spike (high d-spike). **(B)** Bar graphs of the slope **(Bi)** and amplitude **(Bii)** of measured EPSPs. Numbers in the boxes indicate cells tested. Error bars represent SEM. **p < 0.01.

**Figure 2:**
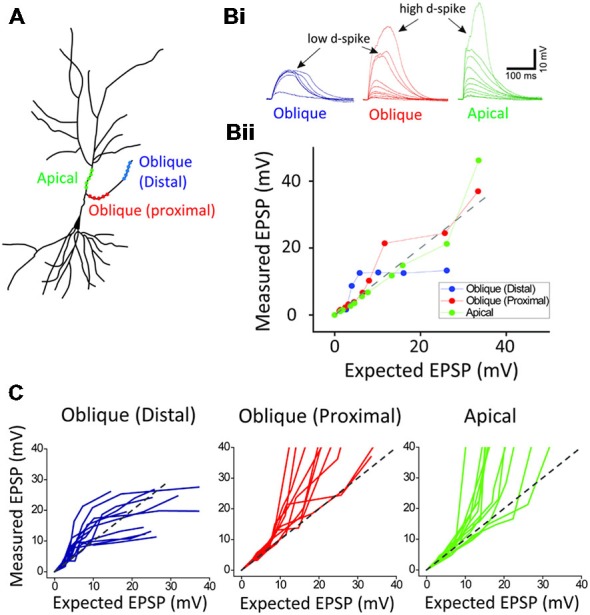
**Location-dependent two distinct dendritic spikes. (A)** a schematic of photoactivated three locations (distal and proximal oblique, and apical trunk) of a hippocampal neuron. **(B)** Representative traces and plots of measured EPSP upon the activation of distal and proximal oblique, and apical trunk. **(C)** Population data. Each photoactivation of distal oblique and apical trunks produces the low- and high-threshold dendritic spikes, respectively, while the photoactivation of a proximal oblique elicits both the low- and high-threshold dendritic spikes.

**Figure 3:**
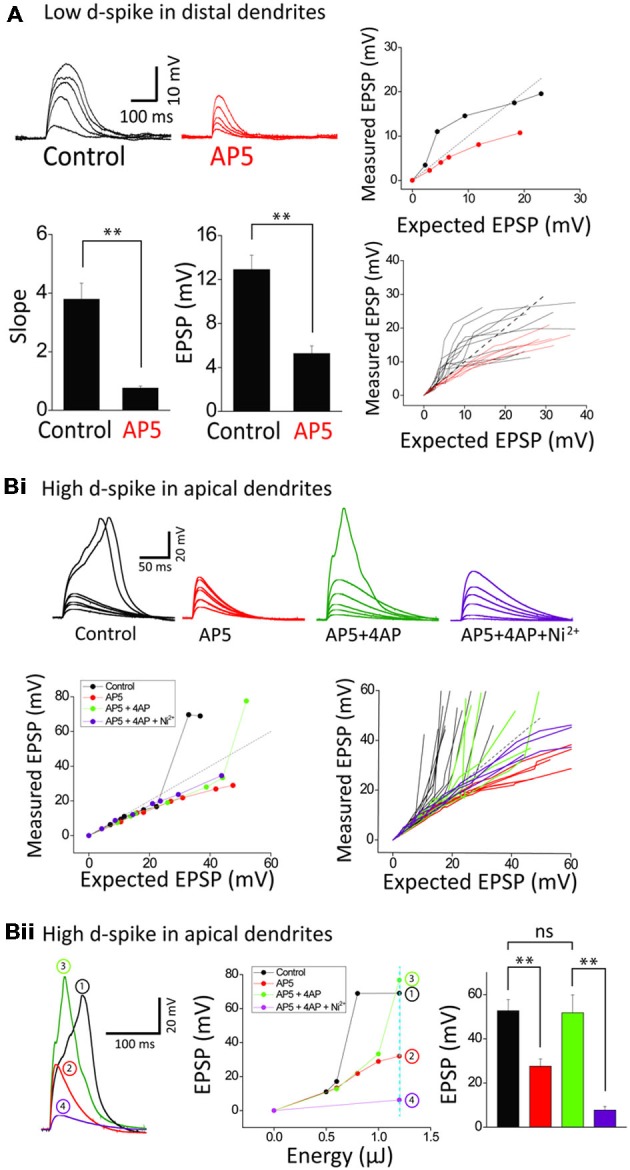
**Ionic composition of two distinct dendritic spikes. (A)** Representative traces and plots illustrating the responses of control and +AP5 (100 μM) in a distal dendrite. AP5 application causes transformation of integration property from supra- to sub-linearity. Bar graphs of the slope and amplitude of measured EPSPs. Numbers in the boxes indicate cells tested. **(Bi)** Representative traces and plots illustrating the responses of control, +AP5, +4AP (3 mM) and +Ni^2+^ (1 mM) in an apical dendrite. Note that the high-d spikes eliminated by AP5 are restored by 4AP application. **(Bii)** Representative traces illustrating the responses of control, +AP5, +4AP and +Ni^2+^ at the dotted line in the EPSP amplitude plot as a function of energy. Shown are that the high d-spike is mediated by I_ca_ channels of an apical trunk which is commonly suppressed by 4AP-sensitive I_A_ channels. Statistics are performed in the plot of peak responses to nearly maximum stimulus intensity. Error bars represent SEM. **p < 0.01.

The procedures for 3D digital holographic photolysis have been described previously in detail (Yang et al., 2011, 2014b). Unlike conventional uncaging techniques,the holographic method permits glutamate photolysis to be directed precisely at multiple x-y-z dimensions simultaneously. Briefly,the holographic beam was brought into the optical axis of an upright fluorescence microscope (Olympus BX51) below the epi-fluorescence unit,with a long-pass dichroic mirror. The output beam of a 150 mW, 405 nm diode laser (CNI Laser) is expanded by a beam expander (3X) to fill the short axis of a reflective spatial light modulator (SLM; LCOS Hamamatsu, model X10468-05). The SLM plane is projected onto the back aperture of the microscope objective through a telescope (L1, f1 = 750 mm; L2, f2 = 500 mm). The magnification of the telescope is chosen in order to match the SLM short axis with the diameter of the objective’s back aperture (Olympus, 60× W 0.9NA). The undiffracted component (zero order spot) is removed by placing a small (<0.5 mm) anodized metal plate on antireflective coated glass plate at the focal plane of L1. The algorithm for the phase hologram calculation and calibration of the temporal spatial resolution were previously described (Yang et al., 2011). Holographic stimulation has the resolution to target individual dendritic spines (Nikolenko et al., 2008; Yang et al., 2011). The spot size is previously measured optical resolution of 0.4 and 2 μm in the transverse and axial directions, respectively (Yang et al., 2011). The effective resolution for photolysis of this single photon system has been previously documented to be 3.1 and 8.7 μm in the transverse and axial direction. Two strategies have been used for data analysis regarding dendritic integration: sequential addition of dendrite spots and increasing laser power on the same spots. The sequential addition of spots was intended to measure the summation property (e.g., supralinear or sublinear for Figure 1 slope, Figures 2, 3A slope, Figures 3Bi, 4) while the stimulation protocol with increasing laser power has been used for measuring the actual amplitude over a wide range of stimulation intensity (i.e., reduction of EPSP amplitude for Figures 1, 33A EPSP(mV), Figures 3Bii, 5. All data were shown as mean ± standard error (SEM). An unpaired *t*-test between groups and paired *t*-test within a group upon drug application were done (significance, **p < 0.01).

**Figure 4:**
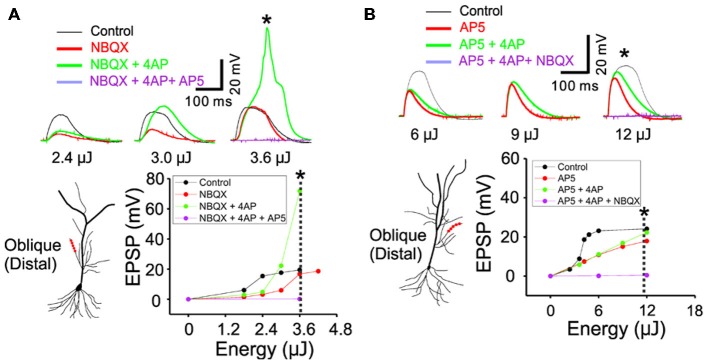
**The inactivation of I_A_ by 4AP recruits the active conductance of NMDARs but not the AMPARs. (A)** The representative traces illustrating the responses of control, +NBQX (20 μM), +4AP (3 mM) and +AP5 (100 μM) at three input strength of weak (2.4 μJ), middle (3.0 μJ) and strong (3.6 μJ) energy. The focal photolysis of caged glutamate at the distal tip of an oblique dendrite elicits potentials (*black traces*). Blocking the AMPA component of the response increases the threshold for the potentials (*red*). Additional blockade of I_A_ evokes the potentials even at moderate stimulus intensity and elicits a calcium spike at higher intensities (*green*). Blockade of both the AMPA and NMDA components results in no observable response to the photostimulation (*purple*). * Marks the traces associated with the dotted line in the plot of peak responses to stimulus intensity. **(B)** The representative traces illustrating the responses of control, +AP5, +4AP and +NBQX (20 μM) at three input strength of weak (6 μJ), middle (9 μJ) and strong (12 μJ) energy. It is notable that, compared to the blockade of NMDARs, additional blockade of I_A_ exerts little or no effect on the AMPA mediated potentials (AP5 *red vs.* +4AP *green*).

**Figure 5:**
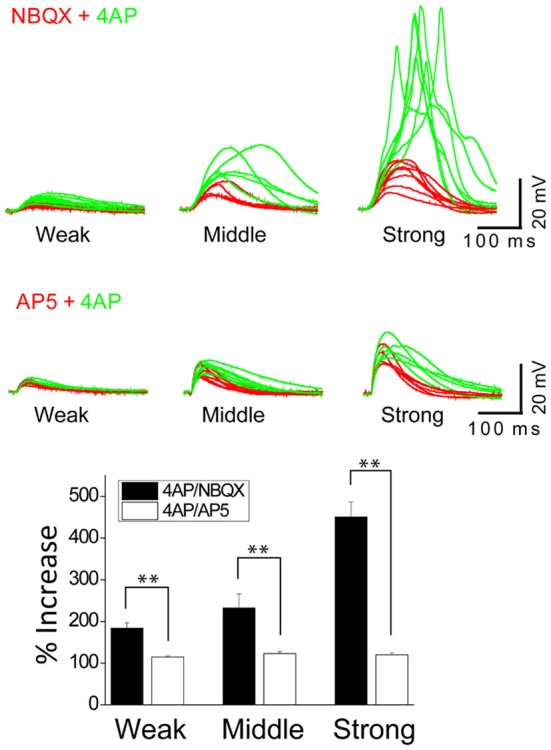
**The inactivation of I_A_ by 4AP potentiates the activation of NMDARs but not AMPARs**. The top traces illustrate AMPAR-mediated EPSPs in a thin oblique dendrite over a wide range of input strength; weak (averaged 3.7 μJ), middle (averaged 6.3 μJ) and strong (averaged 11.2 μJ) energy (*n* = 6). The middle traces illustrate NMDAR-mediated EPSPs in a thin oblique dendrite over a wide range of input strength; weak (averaged 2.2 μJ), middle (averaged 2.4 μJ) and strong (averaged 4.3 μJ) energy (*n* = 6). Shown are population data for % increase of EPSP amplitude after the addition of 4AP. SEM. **p < 0.01.

### Pharmacological Agents

Concentrated stock solutions of various agents were prepared and diluted a final concentration before use. For uncaging experiments, MNI-caged-L-glutamate (Tocris, Ellisville, MO, USA) were prepared fresh each day at final concentration in physiological solution. All agonists and antagonists were purchased from Sigma (St. Louis, MO, USA) or Tocris (Ellisville, MO, USA). The presence of tetrodotoxin (TTX, Tocris) is provided for each experiment.

## Results

### Two Distinct Location-Dependent Dendritic Spikes

Whole-cell patch recordings were made from CA1 pyramidal neurons, with visualization of the dendritic arbor through Alexa 594. When oblique dendrites became visible, the 3D coordinates of the photolysis sites on the dendrites were determined. Tetrodotoxin (TTX, 1 μM) was added to examine only Ca^2+^ delivering channel-medated dendritic spikes. In a single dendrite, one uncaging spot was first stimulated and then more spots (7–10 spots) were sequentially added. At first, the photolyzed uncaging responses summed linearly at a few uncaging sites (Figure 1). Upon the increasing number of the uncaging spots, a supralinear potential appeared, namely a low-threshold dendritic spike (*low d-spike*). The averaged slope and depolarization immediately before the onset of the low d-spike was 1.1 ± 0.1 and 4.5 ± 0.7 mV, respectively. The averaged slope and depolarization of low d-spike was 3.5 ± 0.5 and 15.2 ± 1.5 mV, respectively (Figure 1, * low d-spike; n *= 12). When the number of photolyzed spots was further increased, summation became more supralinear, thereby leading to a high-threshold dendritic spike (*high d-spike*). The slope immediately before the onset of the high d- spikes was 0.9 ± 0.2. The averaged slope and depolarization of high d-spike was 6.3 ± 0.9 and 51.4 ± 1.8 mV, respectively (Figure 1, *high d-spike; n* = 12).

Next, in order to investigate if there is the location dependency for the low and high d-spikes, we separately photolyzed three regions: distal oblique, proximal oblique, and apical trunk (Figure 2A). The photostimulation of a distal oblique segment (100 μm away from a branch point, *n* = 12) elicited only the low d-spikes without the high d-spikes even at nearly maximum laser power (e.g., 12 μJ; Figure 2B; *distal blue*) whereas the photoactivation of a proximal dendrite near an apical trunk produced both the low- and high d-spikes over the increased photostimulation (Figure 2B, *proximal red; n* = 12). On the other hand, the photolysis of an apical dendrite only elicited the high d-spikes without the pronounced low d-spike (Figure 2B, *apical green; n* = 12). There are characteristic dendritic spikes in these three areas (Figure 2C). These results suggest that the low and high d-spikes hold distinct spatial origins, with distal and somato-apical compartments, respectively.

### Ionic Components of Two Distinct Dendritic Spikes

We next examined the ionic mechanisms underlying the two distinct spikes. The supralinearity displayed in an oblique dendrite is thought to be mediated mostly by the activation of NMDARs (Polsky et al., 2004; Losonczy and Magee, 2006; Major et al., 2008; Yang et al., 2014a). Blocking NMDARs by AP5 (a NMDAR blocker, 100 μM), but not Ni^2+^-sensitive-voltage-gated Ca^2+^ channels (Yang et al., 2014a), disturbed summative supralinearity in distal oblique dendrites in all 12 neurons tested (Figure 3A). The slope and amplitude of low d-spikes is blocked by AP5 (*n* = 6; Control: 3.8 ± 0.5, 12.9 ± 1.3 mV; AP5: 0.8 ± 0.1, 5.3 ± 0.7 mV, *p* < 0.01, paired *t*-test). The eliminated supralinearity by loss of NMDAR function was rescued by inactivation I_A_ by 3 mM 4AP (see Figure 4A for the details). On the other hand, the high d-spikes were considered to be mediated by Ca^2+^ spikes including NMDA spikes (Larkum et al., 2009). As expected, AP5 application eliminated the high d-spikes in the apical trunk (Figure 3Bi, *red*). Furthermore the additional inactivation of I_A_ by 4AP, an A-type voltage-gated K^+^ channel blocker, rescued the high d-spikes in the apical trunk, although they appeared either at more uncaging spots (Figure 3Bi) or higher energy per spots (Figure 3Bii) than the control condition. This result suggests that high d-spikes can be also generated by other Ca^2+^-permeable channels besides NMDARs, and are actively suppressed by I_A_, in the apical trunk. Together with AP5 and 4AP, subsequent application of Ni^2+^ (1 mM) eliminated the remnant high d-spikes (Figure 3B, *purple*), suggesting the coordination of Ni^2+^ sensitive-voltage-gated Ca^2+^ channels together with NMDARs in the generation of high d-spikes. We then tested the response amplitudes over a wide range of laser power intensity in the apical trunk; in this design, it reveals how much the absolute response amplitude was affected by each blocker. AP5 application decreased the amplitude of the high d-spikes and Ni^2+^ application further dampened the amplitude (Figure 3Bii). In summary, these results show that there are two different dendritic spikes having differential ionic composition, both of which were shaped by opposing action of Ca^2+^-permeable channels and I_A_.

### I_A_ is a Functional Partner of NMDA Receptors but not AMPA Receptors

In order to study the interaction between I_A_ and NMDARs, we first set out to isolate the NMDAR-mediated component with bath application of 20 μM NBQX, an AMPAR antagonist. Photostimulation was directed at the distal oblique dendrite to produce only the low d-spikes (Figure 4A, *black*) which were known to be mediated by only NMDARs but not Ni^2+^ sensitive-voltage-gated Ca^2+^ channels (Figure 3A; Yang et al., 2014c). Application of 4AP had little or no effect on NMDAR-mediated potentials at the low stimulus intensity (Figure 4A; 2.4 μJ, *red vs. green*). At the middle stimulus intensity (3.0 μJ), however, the addition of 4AP induced a response similar to the low d-spikes (Figure 4A; 3* μ*J, *green*). When the condition was set at the high stimulus intensity, 4AP produced a high d-spike (Figure 4A; 3.6 μJ, *green*). Next, the AMPAR-mediated potentials were isolated in the presence of AP5 (Figure 4B, *red*). Under this condition, 4AP was added to investigate if there is an interaction of AMPARs with I_A_. There was a negligible change of the AMPAR response upon addition of 4AP even at higher laser intensity (Figure 4B; 6~12 μJ, *red vs. green*). Population data for percent increase of EPSP amplitude after the addition of NBQX and 4AP were shown in Figure 5 (Weak: 114.8 ± 2.8% (AMPA) vs. 184.0 ± 12.7% (NMDA); Middle: 122.9 ± 4.9% (AMPA) vs. 232.4 ± 33.2% (NMDA); High: 119.9 ± 4.7% (AMPA) vs. 450.6 ± 36.5% (NMDA), *n* = 7, *p* < 0.01, unpaired *t*-test). It appears that 4AP triggers d-spikes through the activation of Ni^2+^ sensitive-voltage-gated Ca^2+^ channels. In this test, we confirmed that I_A_ can be an internal controller of the voltage-gated Ca^2+^ channels-mediated d-spikes as well as the NMDAR-mediated d-spikes, as also shown in Figure 3B. Taken all together, these results suggest that I_A_ functionally opposes the generation of Ca^2+^ spikes.

## Discussion

In addition to previous studies suggesting a suppressive role of I_A_ in back-propagating APs, our study further demonstrated a critical role of I_A_ as a counterpart to the slow-acting Ca^2+^ channel-mediated potentials in anterograde dendritic spikes. The photoactivation of an oblique dendritic branch produced two distinct dendritic spikes: (1) NMDAR-mediated low d-spikes in thin branch and (2) NMDAR- and VGCC-mediated high d-spikes in apical trunk. Such spikes were controlled by the interaction between Ca^2+^-permeable channels and I_A_ channels; in principle, I_A_ currents appeared to suppress Ca^2+^-mediated excitability. This result proposes a potential dendritic mechanism underlying the neuronal hyperexcitability driven by the loss of I_A_.

### A Counterbalancing Role of I_A_ on NMDAR-Mediated Spikes

What would be the logic of using the fast activating and inactivating conductance of I_A_ to control the slow-acting NMDAR-mediated spikes? The fast activation of I_A_ can provide precisely timed suppression of the slower NMDAR response, because I_A_ exerts a stronger inhibitory action prior to the initiation. In this regard, dendritic I_A_ can be an effective form of fast feed-forward inhibition. Regenerative conductance of VGCCs and NMDARs can slip easily towards depolarization in response to background activity. Even subtle changes in the activity of these channels may cause excessive neuronal excitability. For this, an active filter is required to efficiently suppress the regenerative conductance-mediated hyperexcitability. Because the active filter minimally affects non-regenerative conductances such as fast-acting AMPAR-mediated EPSPs (see Figure 4), it could allow AMPAR-mediated synaptic transmission to propagate. Thus, the properties of dendritic I_A_ seem to fit the description of such an active filter. Here, we postulate that dendritic I_A_ creates a selective barrier for NMDA spikes.

### The Role of I_A_ on Brain Diseases

Blockade of A-type K^+^ channels facilitates the spread of depolarization from the stimulated dendrite into adjacent dendrites such that Ca^2+^-mediated potentials in one branch now depolarize adjacent branches (Cai et al., 2004; Grewe et al., 2010). Consistent with this report, our data also support an essential role of I_A_ channels on dendritic compartmentalization. Inactivation of I_A_ is likely to promote forward propagation of dendrite spikes from thin branches to an apical truck. In this regard, I_A_ inhibition can serve as a basic cellular model of epileptic neurons. In fact, drugs that attenuate or downregulate I_A_ activity have been well recognized as pro-convulsants. For example, blockade of I_A_ has been a well-established epilepsy model in animals; downregulation of I_A_ also caused epileptic discharge in an animal model of temporal lobe epilepsy (Bernard et al., 2004; Su et al., 2008). Surgical tissues obtained from patients with hippocampal sclerosis showed decreased immunoreactivity of Kv4.2 in the dendritic region of the hippocampus (Aronica et al., 2009). Considering these previous reports, it is possible that the alteration of I_A_ activity can cause the disruption of normal dendritic excitability and compartmentalization, thereby leading to epileptic discharge.

Fragile X syndrome was also characterized by neuronal hyperexcitability and abnormal sensitivity and plasticity to sensory stimuli (Bureau et al., 2008; Bhakar et al., 2012; Yang et al., 2014c; Zhang et al., 2014). In fmr1 (fragile X mental retardation): (1) knockout-out mice, dendritic excitability was increased and (2) the protein level of Kv4.2 was significantly decreased, both leading to a high incidence of epileptic discharge (Chuang et al., 2005; Gross et al., 2011; Lee et al., 2011). Similar observations were demonstrated in animal models of Alzheimer’s disease. Amyloid beta (Aβ) blocked I_A_ function in pyramidal cell dendrites, which eventually causes an increase in dendritic membrane excitability (Chen, 2005). The simulation study further demonstrated that the oblique branches were especially vulnerable to the increased excitability due to the abnormal activation of I_A_ channels by Aβ (Morse et al., 2010). In summary, the imbalance between Ca^2+^-permeable channels and I_A_ may provide an ionic basis of potential cellular mechanisms underlying several neural diseases.

## Author Contributions

Sungchil Yang, Sunggu Yang and Cha-Min Tang wrote the main manuscript text. Sungchil Yang and Sunggu Yang did all experiments and prepared all figures. All authors reviewed the manuscript.

## Conflict of Interest Statement

The authors declare that the research was conducted in the absence of any commercial or financial relationships that could be construed as a potential conflict of interest.
